# Subgroups of musculoskeletal pain patients and their psychobiological patterns – The LOGIN study protocol

**DOI:** 10.1186/1471-2474-13-136

**Published:** 2012-08-03

**Authors:** Andreas Gerhardt, Mechthild Hartmann, Jonas Tesarz, Susanne Janke, Sabine Leisner, Günter Seidler, Wolfgang Eich

**Affiliations:** 1 Department of General Internal Medicine and Psychosomatics, University Hospital Heidelberg, Im Neuenheimer Feld 410, D-69120, Heidelberg, Germany

**Keywords:** Chronic non-specific musculoskeletal pain, Endocannabinoids, Mental comorbidity, Pain drawing, Pain extent, Quantitative sensory testing, Mechanism-based, Subgroup classification, Nerve growth factor, Trauma

## Abstract

**Background:**

Pain conditions of the musculoskeletal system are very common and have tremendous socioeconomic impact. Despite its high prevalence, musculoskeletal pain remains poorly understood and predominantly non-specifically and insufficiently treated.

The group of chronic musculoskeletal pain patients is supposed to be heterogeneous, due to a multitude of mechanisms involved in chronic pain. Psychological variables, psychophysiological processes, and neuroendocrine alterations are expected to be involved. Thus far, studies on musculoskeletal pain have predominantly focused on the general aspects of pain processing, thus neglecting the heterogeneity of patients with musculoskeletal pain. Consequently, there is a need for studies that comprise a multitude of mechanisms that are potentially involved in the chronicity and spread of pain. This need might foster research and facilitate a better pathophysiological understanding of the condition, thereby promoting the development of specific mechanism-based treatments for chronic pain. Therefore, the objectives of this study are as follows: 1) identify and describe subgroups of patients with musculoskeletal pain with regard to clinical manifestations (including mental co-morbidity) and 2) investigate whether distinct sensory profiles or 3) distinct plasma levels of pain-related parameters due to different underlying mechanisms can be distinguished in various subgroups of pain patients.

**Methods/Design:**

We will examine a population-based chronic pain sample (n = 100), a clinical tertiary care sample (n = 100) and pain-free patients with depression or post-traumatic stress disorder and pain-free healthy controls (each n = 30, respectively). The samples will be pain localisation matched by sex and age to the population-based sample. Patients will undergo physical examination and thorough assessments of mental co-morbidity (including psychological trauma), perceptual and central sensitisation (quantitative sensory testing), descending inhibition (conditioned pain modulation, the diffuse noxious inhibitory control-like effect), as well as measurement of the plasma levels of nerve growth factor and endocannabinoids.

**Discussion:**

The identification of the underlying pathophysiologic mechanisms in different subgroups of chronic musculoskeletal pain patients will contribute to a mechanism-based subgroup classification. This will foster the development of mechanism-based treatments and holds promise to treat patients more sufficient.

## Background

Chronic pain conditions of the musculoskeletal system are common and of high socioeconomic relevance [1-4]. This is especially true for pain conditions with widely unknown pathogeneses, such as non-specific chronic back pain (CBP), chronic widespread pain (CWP), and fibromyalgia syndrome (FMS). In addition, the prevalence of these conditions and the demand for consultation and treatment have increased over recent years [5,6], which results in high direct and indirect costs [1,3,7,8].

However, therapeutic approaches in chronic musculoskeletal pain patients are often of minor success [9-16]. This is likely because the aetiology and pathogenesis of chronic musculoskeletal pain are still widely unknown. As a result, treatment for this condition involves predominantly unspecific interventions, although the group of chronic musculoskeletal pain patients is believed to be heterogeneous [17,18]. Differences in response to the same treatment in patients with the same disease could be explained by different underlying mechanisms contributing to the generation and maintenance of pain [19,20]. The situation is complicated by the finding that the same disease can derive from various pathophysiological mechanisms. Conversely, the same pathophysiological mechanism may be of interest in distinct diseases [20].

The heterogeneity is supported by strong hints that subgroups exist that differ in terms of aetiopathology, clinical symptomatology, and psychophysiological patterns. A recent study revealed distinct somatosensory profiles in CBP and FMS: FMS patients showed increased sensitivity for different pain modalities in all measured body areas, which suggests central disinhibition (or a deficient pain inhibitory system) as a potential mechanism. CBP subjects, in contrast, exhibited localised alterations within the affected segment. Such alterations may be due to peripheral sensitisation [21]. This finding is in accordance with the main hypothesis of a mechanism-based diagnosis in chronic pain syndromes, which proposes that defined symptoms and signs reflect possible underlying neurobiological pain mechanisms [19,22]. Consequently, these subgroups should be treated with specific mechanism-based approaches, but to date, they have been treated with the same non-specific multimodal treatment programs. Therefore, the assessment of chronic pain and research identifying various factors associated with the development, maintenance, and spread of chronic pain, including their neurobiological correlates, is highly relevant.

Chronic pain has been found to be associated with a higher prevalence of mental co-morbidity. Patients with CBP [23,24], CWP [25], and FMS [26] suffer from mental disorders significantly more often than pain-free controls. This finding is especially true for anxiety disorders and mood disorders, which were found to have prevalence rates of 20.9% and 12.7%, respectively, in a population-based sample of patients with chronic back pain [23]. Of further interest is the role of psychological trauma, which has been neglected in previous research. Traumatic events have higher prevalence rates in patients with pain compared to pain-free controls or patients with other diseases [27-29]. Concerning traumatic experiences, it was suggested that multiple traumas have a cumulative effect on physical health, including back pain and that the impact of the trauma on health may be independent of post-traumatic stress disorder (PTSD) symptomatology [30,31].

The assessment of chronic pain and mental comorbidity on a psychobiological basis may detect common underlying pathophysiological changes. With regard to pain processing there are studies that suggest a role for central disinhibition mechanisms in depression and, to a lower extent, in patients with FMS compared to healthy controls [32]. Alterations in pain processing among patients with depression or FMS were reported previously, but this study found that hyperalgesia was more pronounced in patients with FMS than in those with depression [33]. In patients with FMS with comorbid depression or anxiety, pain processing was not altered in comparison to patients with FMS alone [34]. Thus there seems to be an association of chronic pain or depression with altered pain processing, although chronic pain and comorbid depression did not interact with pain processing. 

In regard to anxiety disorders and the neglected role of trauma, a study by Defrin et al. described a unique sensory profile of hyposensitivity to non-noxious stimuli, accompanied by hypoalgesia to at-pain-threshold noxious stimuli and hyper-reactivity to suprathreshold noxious stimuli in patients with PTSD and chronic pain compared with healthy controls [35].

This pattern clearly differs from other patient groups with chronic pain, such as those with fibromyalgia, who tend to exhibit pain hypersensitivity [21,36], and from alterations in PTSD, in which context a decreased sensitivity to painful stimuli has been reported [37,38]. The results reported by Defrin et al. appear to be a hybrid of what has been found in pain-free PTSD patients and PTSD-free pain patients: decreased sensitivity to non-painful stimuli and increased hyperreactivity to painful stimuli. Sensory processing in anxiety disorders other than PTSD is believed not to differ from processing in healthy controls [35]. Another aspect of the psychobiology of pain is pain inhibition. It was found that pain inhibition is deficient in FMS patients but normal in those with depressive disorder [33]. Another study reports evidence that pain inhibition in FMS is more pronounced in patients with comorbid depressive symptoms compared to those with FMS alone [39]. However, due to heterogenic sample selection and different testing methods, the results in regard to pain processing and pain inhibition in chronic pain and mental disorders are inconsistent and partially contradictory [40]. Therefore, a comprehensive measurement of the clinical manifestation and psychobiological aspects of chronic pain is necessary.

**Figure 1:**
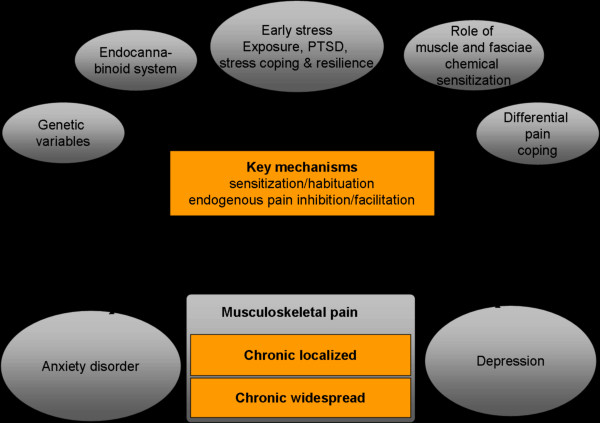
Theoretical framework of our project.

To challenge the topic of a mechanism-based subgroup classification of chronic pain patients and to establish specific mechanism-based treatments [41], further variables of interest must be considered to guarantee a more holistic approach, compared to that pursued in prior research. Therefore, we developed a theoretical framework (Figure 1), which investigates the role of chemical sensitisation (nerve growth factor; NGF) [42-46], the endocannabinoid system [47,48], and other psychological variables (e.g. early stress exposure, stress and pain coping, resilience) [49-51] as well as genetic variables [52-54] in addition to mental comorbidity and psychophysiological patterns. NGF is an important key mediator of some forms of persistent pain and plays an important role in the switch from acute to chronic pain as well as the spatial spread of pain [42-46]. The endocannabinoid system refers to a group of neuromodulatory lipids that is relevant for pain memory and pain extinction [47,48]. Accordingly, these variables have proven to be of interest in chronic pain and to be promising in its treatment. In line with that, the current study addresses the association between the clinical manifestation of chronic musculoskeletal pain (including mental comorbidity) and neurobiological changes.

Therefore, the purpose of the present study is to 1) identify and describe subgroups of patients with musculoskeletal pain with regard to clinical manifestation (including mental comorbidity), 2) investigate whether distinct sensory profiles due to different underlying mechanisms can be distinguished in different subgroups of pain patients 3) and to measure plasma nerve growth factor levels and to analyse distinct endocannabinoid profiles in different subgroups of pain patients.

## Methods

This study is part of the consortium ‘Localized and Generalized Musculoskeletal Pain: Psychobiological Mechanisms and Implications for Treatment (LOGIN)’ funded by the German Federal Ministry of Research and Education (01EC1010A-F). More details concerning LOGIN can be found elsewhere [55,56]. This report focuses on subproject number six (SP6) ‘Subgroups Characterised by Psychological Trauma, Mental Co-morbidity, and Psychobiological Patterns and Their Specialised Treatment’. All participants must provide written informed consent before inclusion in the study. The study has been approved by the Ethics Research Committee of the Faculty of Medicine, University of Heidelberg (S-261/2010) and will be carried out in compliance with the Helsinki Declaration.

### Design

The study uses a descriptive and exploratory design. We will include 200 patients with chronic musculoskeletal pain from different settings (a population-based setting and a tertiary care setting) and 90 controls (pain-free patients with PTSD, depression, and healthy controls without mental disorders). All participants will undergo a physical examination. The relevant sociodemographics and measures of clinical manifestations of chronic pain are reported in Table 1: measurements of potential pathophysiological mechanisms are reported in Table 2.

**Table 1:** Variables and methods used to assess clinical manifestations of chronic non-specific musculoskeletal pain

**Questionnaires**	**Variables**
Chronic Pain Grade Questionnaire (CPG)* [65,66]	Severity of chronic pain problems (disability, pain intensity)
Pain Experience Scale (SES) [64]	Sensory and affective descriptors of pain
12-Item Short-Form Health Survey (SF-12)* [74,75]	Health-related quality of life
Resilience Scale (RS11)* [97,98]	Resilience (personal competence, acceptance of self and life)
Hospital Anxiety and Depression Scale (HADS-D)* [99,100]	Anxiety and depression
Childhood Trauma Questionnaire (CTQ)* [72,73,101]	Childhood and adolescence maltreatment (physical and emotional abuse, sexual abuse, physical and emotional neglect)
Pain drawing (pain location) [62][63][6]	Perceived location(s) of pain will be assessed using digitised pain drawings. Classification into categories of chronic local and chronic widespread pain.
Sociodemographics (self-report questions)	Age, sex, marital status, education, employment status
**Interviews**	
Structured Clinical Interview for DSM-IV Axis I Disorders + Axis II (SCID I + II)* [69]	DSM-IV Axis-I and Axis-II mental disorders
**Physical examination**	
ACR Criteria for Fibromyalgia (ACR Classification) [62][102]	Tenderpoint count and documentation of specific symptoms
Physical Impairment Scale (PIS) [67]	Physical impairment (total flexion, total extension, average lateral flexion, straight leg raising, spinal tenderness, bilateral active straight leg raising, and sit-up)
Back Performance Scale (BPS) [68]	Disability. Tests of daily activities (Sock Test, Pick-up Test, Roll-up Test, Fingertip-to-Floor Test, and Lift Test)

* German Version.

**Table 2:** Methods used to assess the potential mechanisms involved in chronic non-specific musculoskeletal pain

**Measures**	***Variables***
Quantitative Sensory Testing (QST) [76,103]	Comprehensive profiles of somatosensory functions (thermal and mechanical detection and pain thresholds, vibration thresholds, and pain sensitivity to sharp and blunt mechanical stimuli). Discrimination between local vs. generalised and peripheral vs. central nervous mechanisms.
Conditioned Pain Modulation (CPM, the diffuse noxious inhibitory control-like effect) [77,78]	A descending pain inhibitory mechanism that inhibits nociceptive activity arising from the afferent primary fibres at multiple levels of the dorsal horn, resulting in diffuse pain inhibition. These descending pain pathways originate from the brainstem and have significant inhibitory actions on nociceptive activity, thereby affecting pain perception.
*Blood Tests*	
Nerve Growth Factor (NGF)	Plasma NGF levels (proximity ligand ELISA techniques)
Endocannabinoids + related lipids (ECs)	EC (anandamide (AEA), 2-arachidonoyl glycerol (2-AG), 1-arachidonoly glycerol (1-AG), palmitoyl ethanol amine (PEA), oleoyl ethanol amine (OEA), arachidonic acid) in human plasma (large- scale lipidomic profiling using the LC-MS/MS QTrap ABI5500)
Genetics	2* 9 ml EDTA tubes, stored for the second funding period

### Samples and patient recruitment

We will recruit patients with *non-specific chronic musculoskeletal pain* as well as control subjects that are not in pain: 1) Population-based sample: In a previous *population-based* study (“Generalization of Pain: A prospective population-based survey with clinical examination” as part of the German Back Pain Research Network, supported by the Federal Ministry of Education and Research; [21,23,57,58]), we established a representative sample of patients with chronic local and chronic widespread back-pain. For the present study, 100 patients from this representative study sample will be randomly recruited. 2) Tertiary care setting: We will recruit 100 consecutive musculoskeletal pain patients from the *tertiary care Musculoskeletal Pain Centre* at the University Hospital Heidelberg. 3) Control subjects: To determine whether the results are specific for pain, we will further investigate three groups of *pain-free patients*: a) *PTSD* patients (n = 30), b) patients with *depression* (n = 30), and c) *healthy controls* (n = 30). Patients with PTSD and depression will be recruited in our Psychosomatic Outpatient Centre at the University Hospital of Heidelberg. Healthy controls will be recruited by flyers posted around the local community. All groups will be matched with respect to age, sex, and (if appropriate) pain location to our population-based sample. Thus, we will include at least 200 patients with non-specific chronic pain and 90 pain-free subjects.

### Inclusion and exclusion criteria

The *inclusion criteri*a for pain samples are non-specific chronic musculoskeletal pain lasting for ≥ 45 days during the past three months, at least 18 years of age, and fluent German language skills. All control participants (participants with PTSD, depression, and healthy controls) should be pain-free. Because the point prevalence of back pain in the German population was more than one third and the 1-year prevalence was higher than 75% [57], the recruitment of patients that were absolutely pain free within the last three months, will not be feasible and will not reflect reality. Therefore, we aim to recruit only absolutely pain-free participants. If this is not possible, we will define pain-free as follows: 1) less than one day (< 24 hours) spent in pain per week within the last three months. 2) Pain intensity < 3 on an 11-point numeric rating scale on the days when the patient is in pain. 3) Pain does not interfere with normal activities or work. These criteria are adapted from standardised definitions of back pain [59] and its recurrence [60]. Participants must also be pain free on the day of participation in the study. Patients who have a previous history of chronic pain will be excluded. Participants with PTSD and depression must fulfill DSM-IV diagnoses of the respective mental disorder. Patients with PTSD must be free of affective disorders, and patients with depression must be free of anxiety disorders. Healthy controls are not allowed to meet any DSM-IV diagnosis. The *exclusion criteria* are specific pathologies of CBP (e.g., spinal canal stenosis, disc hernia, spondylolisthesis, infection, malignancy, rheumatic and systematic inflammatory disorders, and fracture), sciatica pain ≥ than back pain, diseases affecting sensory processing (diabetes, alcohol or substance abuse, neuropathy, inflammatory diseases), pain or surgery at the dorsum of the hand or back surgery in the past three years (because the hand und back are to be subjected to investigation), and cognitive impairment.

### Procedure

#### Chronicity of pain

The number of painful days in the last three months will be determined by a questionnaire and discussed with a physician to rule out misunderstandings. To be classified as suffering from chronic pain, the subject must report experiencing back pain on ≥ 45 days in the last three months.

#### Clinical examination

To verify the inclusion and exclusion criteria, all participants will be questioned about their past medical history and about co-morbidities (neuropathy, diabetes, relevant alcohol consumption, infections, inflammatory diseases, disc hernia, previous severe injuries). Patients will also receive a physical examination (general, rheumatological, orthopaedic, and neurological), including blood tests (and if indicated further technical investigations such as x-ray or MRI) with special attention paid to findings that indicate a specific origin of back pain. Therefore, the “red flags” (hints of the presence of serious pathology according to the Agency for Health Care Policy and Research Low Back Guidelines) and yellow flags will be considered [61], and former medical reports and discharge letters will be taken into account whenever available. In the case of signs of serious pathological findings, participants will be excluded, and a further investigation will be advised. Painful tender points will be identified by tenderness examination using ACR criteria [62].

#### Measures of clinical manifestation

The clinical manifestations of pain will be considered using the pain dimensions (pain intensity, pain location/ extent, pain quality, and pain affect), disability/ impairment (subjective as well as objective measures), and psychological measures (mental comorbidity, early life stress, health-related quality of life, and resilience). Patients will be clustered in homogeneous groups according to their clinical manifestation. In subsequent analyses, we will test whether the clinical manifestation corresponds with specific mechanisms (see below).

#### Pain dimensions

There are at least four dimensions of pain experience that can be distinguished. These are intensity, location, quality, and affect. *Pain intensity*: Pain intensity is defined as how much a person hurts. It will be measured using a numerical rating scale, ranging from 0 ‘no pain’ to 10 ‘worst pain imaginable’. *Pain location*: Pain location can be defined as the perceived location(s) of pain sensations that patients have on or in their body. Spatial distribution patterns (local vs. referred pain) will be assessed using a digitised pain drawings [63]. Moreover, categorisation as CLP, CWP, and FMS will be based on the ACR criteria [62] and a more precise definition elaborated by Harkness et al. [6]. Therefore, each participant will be asked to complete a body pain diagram, marking all areas where pain is experienced. Afterwards, the pain diagram will be discussed jointly by the participant and the physician to rule out any misunderstandings. *Pain quality and pain affect*: Pain quality refers to the specific physical sensations associated with pain. Pain affect is the degree of emotional arousal caused by the sensory experience of pain. The affective and sensory dimensions of pain will be measured using the Pain Experience Scale (SES). The SES is the standard instrument of the German chapter of the International Association for the Study of Pain. The SES consists of 10 items on a sensory subscale (e.g., ‘throbbing’, ‘wrenching’ or ‘stinging’) and 14 items on an affective subscale (e.g., ‘exhausting’, ‘fearful’, or ‘unbearable’). The response format is a four-stage format (0 ‘not appropriate’; 1 ‘somewhat appropriate’; 2 ‘generally appropriate’; 3 ‘fully appropriate’). The sensory score of the SES is the mean of all sensory items; the affective score of the SES is the mean of all affective items. The retest-reliability of the SES lies between .89 and .96, and Cronbach’s Alpha lies between .72 and .92 [64].

#### Disability/ impairment

##### Chronic pain grade (CPG)

The CPG assesses the severity of chronic pain problems. It measures pain intensity and disability in regard to work and daily activities via patients’ self-reports. The CPG comprises 6 items that can be answered on an 11-point numerical rating scale ranging from ‘0’ to ‘10’. The number of days during which the patient experienced a disability during the past three months is assessed. Pain severity can be graded in four hierarchical classes (Grade I, low disability – low intensity; Grade II, low disability – high intensity; Grade III, high disability – moderately limiting; Grade IV, high disability – severely limiting). The CPG has proven reliability (α = .82) and validity [65,66]. To objectify impairment and disability, we will use the Physical Impairment Scale and the Back Performance Scale.

##### Physical impairment scale (PIS)

The PIS was developed as a simple and standardised clinical observation to evaluate physical impairment in patients with chronic low back pain. The test battery combines objective physical findings indicating current functional limitations due to pain. It consists of seven tests measuring lower back movement (total flexion, total extension and average lateral flexion as measured with the inclinometer), straight leg raises, spinal tenderness and strength (bilateral active straight leg raises, sit-ups). The measurements are translated into values of 0 or 1 according to cut-off values and summed. As subjective disability in non-specific low back pain is not explained by anatomic or structural impairment, the PIS measures functional limitation as influenced by the patient’s pain behaviour. The PIS is able to discriminate between pain patients and healthy controls and is related to self-reported disability in the activities of daily living [67].

##### Back performance scale (BPS)

The BPS is an objective clinical assessment tool that can be used to observe self-reported activity limitations in daily functioning caused by lower back pain. The BPS consists of five tests of daily activities (Sock Test, Pick-up Test, Roll-up Test, Fingertip-to-Floor Test, and Lift Test) frequently reported to be limited in back pain patients. Each performance is evaluated by the observer according to operational score definitions and then summed. The five tests are combined to obtain a performance measure of mobility-related activities requiring sagittal-plane mobility. The BPS is able to discriminate between pain patients with different return-to-work statuses and is sensitive to change. Cronbach’s α was .73 [68].

#### Psychological measures

##### Structured clinical interview for DSM-IV (SCID)

To examine the prevalence and the type of mental co-morbidity, the SCID interview, which consists of two parts, will be applied [69]. The SCID is a comprehensive and highly reliable and valid instrument [70]. The SCID-I is a semi-structured interview for the evaluation of major DSM-IV Axis-I diagnoses. With the SCID-I, it is possible to derive both a current and a previous history of psychiatric illness. The SCID-II procedure for assessing personality disorders (PD) is a two-stage process. First, subjects complete a 120-item questionnaire with questions based on the criteria from the DSM-IV. In the second stage, a semi-structured interview is administered. Positive answers must be re-evaluated by the interviewer to diagnose Axis-II PD. According to the SCID-II protocol, we will interview only those subjects who achieve the cut-off (a specified number of positive answers in a specific PD section) on the questionnaire [69]. All SKID interviews will be conducted by two psychologists with graduate training in clinical psychology. To ensure diagnostic reliability, all interviews will be audiotaped. One-fifth of the interviews will be randomly selected and rated by both psychologists. A kappa coefficient will be calculated to assess inter-rater reliability. Both psychologists will conduct 10 SKID interviews in a pilot phase. In cases of low inter-rater agreement further, training will be conducted by an experienced psychiatrist.

The *Hospital Anxiety and Depression Scale (HADS-D)* will be used to determine the severity of anxiety and depression. The HADS-D was especially developed for patients with somatic diseases and thus excludes physical symptoms. Each scale consists of seven items that measure anxiety and depression via the patient’s self-report with a four-stage response format. The HADS-D has good reliability (subscale depression: α = .81; subscale anxiety: α = .80) and validity [71].

##### Childhood Trauma Questionnaire (CTQ

The German Version of the CTQ will be used to measure early stress exposure. The CTQ measures maltreatment during childhood and adolescence and will be applied because it captures factors that are relevant to chronic pain [50] that are neglected by the SKID. The CTQ consists of five subscales (‘emotional abuse’, ‘physical abuse’, ‘sexual abuse’, ‘emotional neglect’, and ‘physical neglect’). Cronbach’s α ranges from .89 to .96, except for the subscale ‘physical neglect’ which yields an α of .62 [72,73].

##### 12-Item short form health survey (SF-12)

The health-related quality of life (HRQoL) will be measured with the SF-12. The SF-12 consists of 12 items on eight scales (‘physical functioning’, ‘role limitations due to physical problems’, ‘bodily pain’, ‘general health’, ‘vitality’, ‘social functioning’, ‘role limitations due to emotional problems’, and ‘perceived mental health’). Response categories vary from 2 to 6 and can be transformed to scale scores ranging from 0 (‘the worst’) to 100 (‘the best’) [74,75].

##### Resilience scale (RS-11)

Resilience is a personality characteristic that moderates the negative effects of stress and promotes adaption. Thus it avoids any potentially negative effects of stress. Resilience will be measured with the RS-11. The RS-11 comprises two factors – ‘acceptance of self and life’ and ‘personal competence’ – with a seven-point response format ranging from 1 ‘disagree’ to 7 ‘agree’. Thus, scores can range from seven to 77, with higher scores reflecting higher resilience. The RS-11 has very good reliability (α = .91).

#### Sociodemographic variables

Sex, age, education, employment status, marital status and further sociodemographic variables will by captured by a questionnaire.

### Measures of chronic pain mechanisms

We will determine whether the patient’s clinical manifestations of pain correspond with various specific potential pain mechanisms. In our study, potential mechanisms are captured through quantitative sensory testing (QST), the evaluation of conditioned pain modulation (CPM, the diffuse noxious inhibitory control-like effect), and analyses of nerve growth factor (NGF) plasma levels and endocannabinoid (ECs) profiles. Such potential mechanisms include peripheral sensitisation, central sensitisation, disinhibition, allodynia, and endogenous descending pain modulation.

#### Psychophysiological mechanisms

##### Quantitative Sensory Testing (QST)

Somatosensory function will be assessed using the comprehensive *QST* protocol developed as part of the German Research Network on Neuropathic Pain (DFNS). Seven tests measuring 13 parameters (warm detection threshold, cold detection threshold, thermal sensory limen, paradoxical heat sensation, cold pain threshold, heat pain threshold, mechanical detection threshold, mechanical pain threshold, mechanical pain sensitivity, dynamic mechanical allodynia, wind-up ratio, vibration detection threshold, and pressure pain threshold) [76] will be conducted. QST testing covers all relevant aspects of the somatosensory system including large and small fibre function as well as signs of central sensitisation (dynamic tactile allodynia, punctate mechanical hyperalgesia). This way, detailed profiles of somatosensory function will be obtained for the tested body areas. The test sites will be distributed throughout the paraspinal muscles (5 cm ± 0.5 cm next to the midline on the autochthon back muscles [L1 to S1]) and on the dorsum of the ipsilateral hand.

##### Conditioned pain modulation (CPM)

Inhibitory pain-modulating mechanisms will be assessed using the CPM, a diffuse noxious inhibitory control-like effect [77-79]. The difference in pressure pain threshold (PPT) before and after the induction of DNIC by phasic heat pain (PHP) will be measured. The appropriate temperature for PHP will be determined by measurement of the heat pain threshold (HPT). The PHP will oscillate ± 1°C around the PHP-temperature. The ratings of PHP pain intensity will be assessed using a computerised Visual Analogue Scale (VAS). The HPT will be obtained using ramped stimuli (1°C/s, 32°C baseline, 0°C and 50°C cutoffs, 8 cm^2^ thermode), which will be terminated when participants press a button. The mean of three consecutive measurements will be calculated. The PPT will be calculated as the mean of three consecutive measurements over the paraspinal muscles (5 cm ± 0.5 cm next to the midline on the autochthon back muscles [L1 to S1]; site contralateral to the QST).

#### Neurobiological measures

##### Nerve growth factor (NGF)

NGF levels in human blood samples will be determined using proximity ligand Elisa techniques. *Endocannabinoids (ECs)*: EC (anandamide (AEA), 2-arachidonoyl glycerol (2-AG), 1-arachidonoly glycerol (1-AG), palmitoyl ethanol amine (PEA), oleoyl ethanol amine (OEA), arachidonic acid) analyses of human blood samples will be performed by large-scale lipidomic profiling using the LC-MS/MS QTrap ABI5500. All analyses of NGF and ECs will be performed in collaboration with our consortium partners.

### Sample size estimation

A sample of more than 200 musculoskeletal pain patients (population-based sample and tertiary care sample) will be acceptable in order to recruit a sufficient number of “cases” with different clinical manifestations (e.g., local vs. generalised pain, different levels of pain affect, anxiety disorders, mood disorders, no mental comorbidity). To estimate the number of patients in different subgroups, we will refer to data from our population-based study. Approximately 61.8% of the patients in our population-based study had chronic local pain (CLP), and 38.2% had chronic widespread pain (CWP). We also found a prevalence of 12.7% for depression and 20.9% for anxiety disorders (using the SCID-I). The prevalence of depression and anxiety may be higher in a tertiary care pain setting, as reported by others [80,81]. Therefore, we expect group sizes that will be sufficient to gather abundant information regarding clinical manifestations. We also consulted recent QST studies and a review regarding DNIC to estimate the required group sizes. For DNIC testing, a systematic review [82] evaluated studies with an average group size of 20. A group size of 20 to 30 is also commonly used in recent QST studies [21,63,83]. We therefore expect our group sizes to be appropriate for the investigation of distinct sensory profiles. Studies investigating endocannabinoids used sample sizes between n = 10 and n = 20 patients per group and reported mean effect sizes between .60 and .80 (e.g. [84,85]). Studies with NGF have reported effect sizes between 2.02 and 4.31 [44,86]. With regard to pain mechanisms, small sample sizes are sufficient to compare subgroups. This will also apply for the groups of pain-free patients with PTSD, depression, and healthy controls (each n = 30, respectively).

### Quality assurance

To ensure that the measurements are reliable and high in quality, the project will have a pilot phase. In this pilot phase the study staff will be trained in the study procedures (if necessary) and conduct paired measurements to ensure reliability and validity. The pilot phase will be finished when the reliability and validity of the measurements has been verified. The study protocols will be tested and adapted if necessary.

### Statistical analyses

Descriptive statistics will be presented with means and standard deviations for continuous variables and absolute numbers and percentages for categorical variables. Questionnaires will be dealt with according to questionnaire manuals. The prevalence of chronic local pain, chronic widespread pain, fibromyalgia, and mental comorbidities will be determined for the population-based sample and tertiary care setting. Explorative cluster analysis will be conducted to establish subgroups based on the clinical manifestations observed. Therefore, the dimensions of pain (see above) and mental comorbidity will be used as cluster variables. Then, we will explore whether different neurobiological profiles (QST profiles, CPM, NGF levels, EC profiles) correspond with these subgroups. Pain drawings will be scanned, superimposed, and transformed into two-dimensional color-coded images. Body areas with high occurrence of pain will be illustrated in dark red; body areas without pain will appear in white. To classify patients who suffer chronic local pain (CLP) or chronic widespread pain (CWP), pain drawings will be analysed according to the ACR criteria [62] and a more precise definition [6]. Quantitative sensory testing (QST) data pre-processing and statistical analysis will be performed according to the protocol established by Rolke et al. [76]. To quantify conditioned pain modulation (CPM), the PPT before PHP will be subtracted from the PPT after PHP. Negative values indicate an analgesic effect due to CPM. Differences between patient groups will be analysed using analyses of co-variance (ANCOVA), followed by Fisher’s least significant difference test. Potential confounders will be included as covariates, if indicated. QST modalities or CPM will be entered as dependent variables, the patient groups as an independent variable. For more detailed information analysing QST data, we will refer to the protocol proposed by Rolke et al. [76]. The same procedure will be applied with regard to nerve growth factor (NGF) and endocannabinoids (ECs).

## Discussion

Establishment of a mechanism-based subgroup classification of pain and the development of specific treatments were suggested almost a decade ago [41]. Since then, the topic has been discussed amid controversy [19,22,87,88]. Small effect sizes of chronic pain treatments were suspected to be due to unspecific treatment approaches, but different pain generating and maintaining mechanisms [19,20]. This possibly is also supported by clinical experience, which shows that the subgroups of chronic pain patients are heterogeneous, even if suffering the same disease like non-specific chronic back pain. However, only a few studies have aimed to identify different pain mechanisms [20,21]. The identification of patient subgroups is needed if we wish to establish distinct pathophysiological mechanisms and targets that are necessary for the development of new analgesic drugs and non-pharmacological mechanism-based treatment options. There is a corresponding lack of evidence for subgroup-specific treatments.

In addition to the identification of specific pathophysiological mechanisms, we will implement a feasibility study that is designed as a randomised controlled trial. We will adapt the proven Eye-Movement-Desensitization-Reprocessing (EMDR) short-time therapy to the subgroup of patients with chronic pain who have experienced psychological trauma. This approach might be promising because EMDR is an effective treatment for patients with PTSD [89,90] or chronic pain [91-94] but has not yet been adapted to patients with chronic musculoskeletal pain who have experienced psychological trauma. However, there are initial signs that this might be a promising approach [95,96]. To identify potential underlying mechanisms, we will use all measurements of our study obtained before and after treatment (plus functional magnetic resonance imaging). Thus, our study will foster the development of new, more specific interventions for chronic pain patients.

The remaining challenge is to match a sign or symptom to a mechanism, but a sign or symptom could potentially be produced by several distinct mechanisms [19,20]. The novel aspect of our research is therefore its comprehensive approach that uses reliable and valid diagnostic tools. This approach comprises many variables that have been shown to be involved in alterations in sensory processing (e.g., mental comorbidity, descending pain modulating systems, nerve growth factor, endocannabinoids). A holistic approach is also needed because research shows that these variables influence each other [35]. The observed alterations might be hybrids of alterations caused by single variables [21,37]. The inclusion of a population-based sample is also reasonable because prior research is usually based on highly selective clinical samples of pain patients, and this might bias research. Notably, the study is part of the LOGIN consortium. LOGIN comprises seven subprojects and includes basic and applied research in animals and humans as well as preclinical and clinical projects. All projects will use a core set of variables that investigates similar pathogenetic mechanisms. This approach enables LOGIN to study aspects in animals that cannot be investigated in humans (e.g., pathophysiological processes in the spinal cord or brain) and to transfer results to the human subprojects and vice versa. This approach will be fostered by the translational aspects of LOGIN. Thus, using the synergy of the different subprojects, the contemporary translation, implementation and dissemination of the results will be guaranteed.

## Competing interest

The authors declare that they have no competing interests.

## Author’s contributions

AG has made substantial contributions to conception and design and has drafted the manuscript. He also participates in data collection, analyses of the data, and coordinates the project. JT has made substantial contributions to conception and design and revised the manuscript critically for important intellectual content. He also participates in data collection. MH and WE have made substantial contributions to conception and design and revised the manuscript critically for important intellectual content. SJ and SL participate in data collection and analyses of the data. GS revised the manuscript critically for important intellectual content. All authors read and approved the final version of the manuscript to be published.

## Acknowledgements

The project is part of the research Consortium LOGIN: “Localized and Generalized Musculoskeletal Pain: Psychobiological Mechanisms and Implications for Treatment” funded by the German Federal Ministry of Education and Research (BMBF, 01EC1010A-E). We thank the members of the consortium LOGIN for their scientific thoughts and constructive discussions in the development of this project. This study is supported by a research grant from the BMBF, project 01EC1010A.

## Pre-publication history

The pre-publication history for this paper can be accessed here:

http://www.biomedcentral.com/1471-2474/13/136/prepub
